# Cognitive abilities are associated with rapid dynamics of electrophysiological connectome states

**DOI:** 10.1162/netn_a_00390

**Published:** 2024-12-10

**Authors:** Suhnyoung Jun, Stephen M. Malone, Thomas H. Alderson, Jeremy Harper, Ruskin H. Hunt, Kathleen M. Thomas, Sylia Wilson, William G. Iacono, Sepideh Sadaghiani

**Affiliations:** Department of Psychology, University of Illinois Urbana-Champaign, Champaign, IL, USA; Beckman Institute for Advanced Science and Technology, University of Illinois Urbana-Champaign, Champaign, IL, USA; Department of Psychology, University of Minnesota Twin Cities, Minneapolis, MN, USA; Institute of Child Development, University of Minnesota Twin Cities, Minneapolis, MN, USA; Neuroscience Program, University of Illinois Urbana-Champaign, Champaign, IL, USA

**Keywords:** Dynamic functional connectivity, Electrophysiology, Hidden Markov modeling, Cognition, Individual differences, Canonical correlation analysis

## Abstract

Time-varying changes in whole-brain connectivity patterns, or connectome state dynamics, hold significant implications for cognition. However, connectome dynamics at fast (>1 Hz) timescales highly relevant to cognition are poorly understood due to the dominance of inherently slow fMRI in connectome studies. Here, we investigated the behavioral significance of rapid electrophysiological connectome dynamics using source-localized EEG connectomes during resting state (*N* = 926, 473 females). We focused on dynamic connectome features pertinent to individual differences, specifically those with established heritability: Fractional Occupancy (i.e., the overall duration spent in each recurrent connectome state) in beta and gamma bands and Transition Probability (i.e., the frequency of state switches) in theta, alpha, beta, and gamma bands. Canonical correlation analysis found a significant relationship between the heritable phenotypes of subsecond connectome dynamics and cognition. Specifically, principal components of Transition Probabilities in alpha (followed by theta and gamma bands) and a cognitive factor representing visuospatial processing (followed by verbal and auditory working memory) most notably contributed to the relationship. We conclude that rapid connectome state transitions shape individuals’ cognitive abilities and traits. Such subsecond connectome dynamics may inform about behavioral function and dysfunction and serve as endophenotypes for cognitive abilities.

## Author Summary

This study investigates the behavioral significance of rapid electrophysiological connectome dynamics features with established heritability. The heritable phenotypes that describe the duration (Fractional Occupancy) and frequency of connectome state switches (Transition Probability) were obtained using hidden Markov model on source-localized EEG data at rest. Using the canonical correlation analysis approach, we found that connectome state transitions unfolding at multiple speeds (e.g., alpha, theta, and gamma) collectively contribute to shape cognitive abilities.

## INTRODUCTION

Cognitive processes are inherently dynamic, and a substantial portion of the dynamic processes underlying cognition unfolds at speeds of a few 100 ms or faster. It stands to reason that ongoing or spontaneous neural processes constituting most brain activity, including large-scale connectome dynamics, affect cognition (Raichle & Gusnard, 2005). Indeed, a great functional significance of large-scale network dynamics for cognitive performance (Kucyi, Tambini, Sadaghiani, Keilholz, & Cohen, 2018; Sadaghiani, Poline, Kleinschmidt, & D’Esposito, 2015) and interindividual differences therein (Eichenbaum, Pappas, Lurie, Cohen, & D’Esposito, 2021; Jun, Alderson, Altmann, & Sadaghiani, 2022) has been established using fMRI. In fact, investigations of functional connectome dynamics and their functional significance have been dominated by fMRI due to its exceptional spatial resolution. Unfortunately, the temporal dynamics captured by the fMRI-derived slow and indirect measure of neural activity, or BOLD signal, misses the rich temporal dynamics that occur on cognitively more relevant subsecond timescales.

These time-varying dynamics in large-scale functional connectivity can be characterized as flexible changes in connectome states, representing the varying strength of connectivity between specific sets of brain regions within the whole-brain connectome, which occur repeatedly over time (Allen et al., 2014; Hutchison et al., 2013). Recently, data-driven approaches, such as hidden Markov modeling (HMM), have been employed to identify temporally recurrent connectome states with state-specific mean and covariance from the observed time series of different brain regions (Baker et al., 2014; Hunyadi, Woolrich, Quinn, Vidaurre, & De Vos, 2019; Quinn et al., 2018; Vidaurre et al., 2016; Vidaurre, Smith, & Woolrich, 2017).

Prior fMRI connectome studies have established that a wide range of behaviors and cognitive processes are linked to such recurrent state dynamics, encompassing both the temporal organization of connectome state transitions (Eichenbaum et al., 2021; Jun et al., 2022; Vidaurre et al., 2017) and changes in the spatial organization of connectivity patterns (Sadaghiani et al., 2015; Shine, Koyejo, & Poldrack, 2016). Particular temporal features of fMRI-derived connectome dynamics, specifically the proportion of the total recording time spent in each connectome state (Fractional Occupancy) and the probability to transition between specific pairs of connectome states (Transition Probability), have not only been linked to behavioral performance (Eichenbaum et al., 2021; Jun et al., 2022; Vidaurre et al., 2017) but also been found to be heritable (Jun et al., 2022; Vidaurre et al., 2017). More specifically, our previous fMRI work has established substantial genetic effects (*h*^2^ ∼ 40%) and behavioral relevance of Fractional Occupancy and Transition Probability (Jun et al., 2022). We further found preliminary evidence for specific genetic polymorphisms predictive of fMRI-derived Fractional Occupancy and Transition Probability via the regulatory impact of modulatory neurotransmitter systems (see our pre-registered work; https://doi.org/10.17605/OSF.IO/VF2ZW). This fMRI-based body of literature establishes that *infraslow* connectome dynamics are endophenotypes driving individually specific cognitive abilities, which, in turn, are heritable (Han & Adolphs, 2020; Jun et al., 2022). Yet, little is known about the functional significance of *rapid* connectome dynamics.

Noninvasive, real-time methods, that is, EEG and Magnetoencephalography (MEG), allow capturing rapid electrophysiological signals with real-time fidelity. Recently, a growing body of work has established the capability to investigate the spatially informative functional connectome and, importantly, its rapid dynamics in source-space with measures addressing source-leakage: static/time-averaged connectome (Brookes et al., 2011; Deligianni, Centeno, Carmichael, & Clayden, 2014; de Pasquale et al., 2010; Hipp & Siegel, 2015; Wirsich et al., 2017), connectome dynamics (Baker et al., 2014; Brookes et al., 2014; Coquelet et al., 2022; Sitnikova et al., 2020; Wirsich, Giraud, & Sadaghiani, 2020); for review, see Sadaghiani and Wirsich (2020). Importantly, we have recently shown that Fractional Occupancy and Transition Probability of rapid connectome dynamics derived from source-space EEG are also under significant genetic influence (Jun et al., 2024). More specifically, we applied HMM to obtain discrete brain states using source-reconstructed resting-state EEG data from two cohorts of twins from the Minnesota Twin Family Study (Iacono, Carlson, Taylor, Elkins, & McGue, 1999; Keyes et al., 2009; Wilson et al., 2019). For each canonical frequency band, we measured temporal characteristics, specifically Fractional Occupancy and Transition Probability, of the HMM-derived brain states. We found that genetic effects explain a substantial proportion of phenotypic variance in temporal (but not spatial) characteristics of connectome dynamics, specifically Fractional Occupancy in beta (44.3%) and gamma (39.8%) bands and Transition Probability in theta (38.4%), alpha (63.3%), and gamma (40%) bands. The substantial heritability of these temporal phenotypes of rapid connectome dynamics led us to question their functional implications for individual differences in cognitive abilities.

In the current study, we focused on the above-described *heritable* time-varying phenotypes of subsecond, electrophysiological connectome dynamics (Jun et al., 2024). These comprised Fractional Occupancy in beta and gamma bands and Transition Probability in theta, alpha, and gamma bands of HMM-derived brain states. Heritable features inherently reflect individual differences and further allow us to narrow the particularly large feature space of rich, electrophysiological connectome dynamics that comprise numerous frequency bands. In 926 individuals from the Minnesota Twin Family Study, we investigated whether these band-specific dynamic phenotypes contribute to interindividual variability in cognitive performance. Specifically, we examined their associations with cognitive task measures using canonical correlation analysis (CCA). To the best of our knowledge, this is the first source-reconstructed EEG study investigating the behavioral significance of rapid temporal connectome dynamics.

## MATERIALS AND METHODS

**Figure 1.**
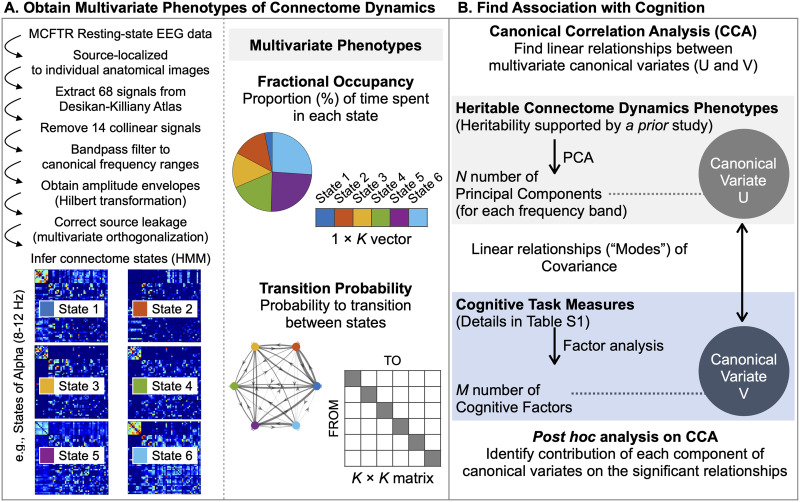
An overview of the analysis pipeline. (A) Source-reconstructed resting-state EEG data entered HMM to extract six discrete connectome states from the amplitude time series of each canonical frequency band. Connectivity matrices reflecting amplitude coupling are shown for each state. These six states are color-coded (blue, red, yellow, green, purple, and light blue) to visually depict their contribution to the connectome phenotypes of interest. We obtained two temporal features of rapid connectome dynamics for each frequency band, namely, the proportion of time spent in each connectome state (Fractional Occupancy) and the probability matrix describing transitions between all possible pairs of discrete states (Transition Probability). For further analysis, we retained Fractional Occupancy and Transition Probability in bands in which they were found to be heritable (Jun et al., 2024). (B) To identify linear relationships (“modes”) between these multivariate phenotypes and cognitive task measures, we conducted CCA on dimensionality-reduced data.

Figure 1 is a schematic representation of the overall approach and analysis subsections. Panel A illustrates how heritable phenotypes of connectome dynamics were derived (cf. Jun et al., 2024). Specifically, we used resting-state EEG signals, which are source-localized to individual anatomical images from the Minnesota Twin Family Study dataset (see the EEG Signal Preprocessing and Source Localization section). The source signals were leakage-corrected and averaged within each of the 68 anatomically distinct brain regions defined using the Desikan-Killiany Atlas (Desikan et al., 2006). Then, we extracted six discrete connectome states from the amplitude time series of each canonical frequency band, using HMM (see the HMM of Connectome States section). These states, represented by amplitude coupling matrices (Figure 1A, bottom left), were associated with a state time course for each subject, indicating the likelihood of each state being active at any given time. From this analysis, we obtained two temporal features of rapid connectome dynamics for each frequency band, namely, Fractional Occupancy and Transition Probability (Figure 1A, right panel; see the Multivariate Temporal Features of the Dynamic Connectome section). These features were constructed in a multivariate manner to comprehensively represent all states. Fractional Occupancy and Transition Probability in specific frequency bands were found to be heritable (Jun et al., 2024) and were moved to the analysis of cognitive associations. Panel B describes the discovery of such associations. Specifically, we conducted CCA (Hotelling, 1936) to identify linear relationships (“modes”) between the multivariate dynamic connectome phenotypes and cognitive task measures. This analysis was carried out on dimensionality-reduced data, encompassing frequency-specific *N* principal components (PCs) of multivariate dynamic connectome phenotypes and *M* cognitive factors (see the Association Between Dynamic Trajectories of States and Cognitive Performance section). Details are provided in the following sections.

### Subjects and Cognitive Measures

Participants for the present investigation are from the two independent cohorts of twins from the Minnesota Twin and Family Research (MCTFR) (Iacono et al., 1999; Keyes et al., 2009; Wilson et al., 2019). Twins in both cohorts have been followed periodically since approximately the age of 11. As part of their most recent assessment, participants underwent structural MRI scans in addition to resting EEG recordings. At time of initial recruitment and at each follow-up, participants gave written informed consent or assent, if under the age of 18, for their participation.

From a total of 1,164 subjects in two independent cohorts of MCTFR twins completing virtually identical assessments, 928 had usable and complete EEG data that were source-localized successfully (see the EEG Signal Preprocessing and Source Localization section), thus permitting HMM-based estimation of discrete connectome states. The included subjects (473 females) were 23–40 years of age at time of data acquisition. Subsequently, 463 sex-matched pairs (926 subjects) were formed for heritability analysis in our previous study (Jun et al., 2024) as follows: 206 monozygotic twin pairs, 112 sex-matched dizygotic (DZ) twin pairs, and 145 pairs of sex-matched unrelated individuals. Each subject entered only one pair. Note that the unrelated individuals are twins whose co-twin lacked complete data and was, therefore, not part of the analytic sample. In the present study, this pool of 926 subjects entered the cognitive association analysis.

We included 15 summary measures from eight cognitive tasks completed as part of a whole-day assessment at the MCTFR. Tasks assessed the following domains: verbal and nonverbal intelligence, visual–spatial processing speed, auditory–verbal learning and memory, auditory–verbal attention and memory, visual–spatial attention and memory, visual–spatial learning and memory, response inhibition, and decision-making under uncertain conditions or risks (see Supporting Information Table S1 for more detailed description for each measure and task). The measures were transformed into *z*-scores. All 926 subjects had more than 50% of cognitive measures (859 subjects with complete dataset).

### MRI and EEG Acquisition

Structural MRI data were collected to build a subject-specific anatomical head model for precise source localization (see the EEG Signal Preprocessing and Source Localization section) on either 3 T Siemens Trio or Prisma MRI scanner (32-channel array head coil) at the Center for Magnetic Resonance Research, University of Minnesota. Three-dimensional T1-weighted sagittal plane anatomical images were acquired using a magnetization-prepared rapid gradient echo sequence with the following parameters: TR = 2,530 ms; TE = 3.65 ms; flip angle = 7°; matrix size = 256 × 256; FOV = 256 mm; GRAPPA = 2; 240 coronal slices with 1-mm isotropic voxels; single shot; interleaved acquisition.

EEG was recorded from 61 scalp electrodes arranged according to the International 10/10 system using a BioSemi ActiveTwo system (BioSemi, Amsterdam, The Netherlands) at 1024 Hz. ActiveTwo amplifiers are Direct-Coupled (DC), and the signals are monopolar. Data were low-pass filtered using a digital fifth-order Bessel antialiasing sinc filter with a cutoff frequency (3-dB attenuation) of 205 Hz. To detect blinks and other eye movements, pairs of electrodes were placed above and below the right eye or on the outer canthus of each eye. Additional electrodes were placed on left and right earlobes, and the average of these signals was derived offline to serve as a reference. As part of the source localization process detailed below, data were re-references to common average reference. During EEG recording, participants rested comfortably in a darkened room, with their head and neck supported while hearing 55-dB white noise played through headphones. Participants were asked to keep their eyes closed and relax. A recorded voice subsequently instructed them to open their eyes or close them at 1-min intervals. A total of 6 min of EEG was collected, 3 min with eyes open and 3 with eyes closed.

### EEG Signal Preprocessing and Source Localization

Preprocessing and source localization are detailed in our prior work (Jun et al., 2024). In brief, using a monitored automated pipeline (https://www.github.com/sjburwell/eeg_commander) of the Minnesota Twin Family Study group and EEGLAB (Delorme & Makeig, 2004) in MATLAB (version R2021b, MathWorks, Inc.), raw EEG data were downsampled to 256 Hz, high-pass filtered above 0.1 Hz, and underwent automated detection of disconnected channels/flat signals, interelectrode electrolyte bridging, large amplitude deviations, and muscle/cap shift (motion) noise. Data were epoched to 1 s, and data that exceeded four normalized median absolute deviations from the median (Rousseeuw & Croux, 1993) in 25% of a 1 s time range or 75% of a given electrode were removed. Among others, this approach is effective in removing periods with head motion artifacts. Ocular artifacts were removed with independent component analysis (ICA) and joint consideration of temporal and spatial signal characteristics.

For source localization, the preprocessed EEG signals were imported into the Brainstorm software (Tadel, Baillet, Mosher, Pantazis, & Leahy, 2011), resampled to 250 Hz, corrected for DC offsets, linearly detrended, and low-pass filtered at 70 Hz. To aid the coregistration of electrode positions and individual subjects’ T1 images, we manually marked fiducial points, including anterior and posterior commissures, interhemispheric point, nasion, and left and right preauricular points. The coregistration was refined by manually moving the electrode positions onto the electrode artifacts visible in the T1 image. We then used the OpenMEEG software (Gramfort, Papadopoulo, Olivi, & Clerc, 2010) with a symmetric boundary element method (BEM) to calculate a forward model of the skull based on subjects’ individual T1 image (Tadel et al., 2019), followed by the Tikhonov-regularized minimum-norm estimation (MNE) inverse method to compute the sources, with default parameter settings for regularization and source depth weighting (Baillet, Mosher, & Leahy, 2001; Tadel et al., 2019).

### Parcellation and Source-Leakage Correction

Preprocessing and source localization are detailed in our prior work (Jun et al., 2024). In brief, we conducted individual cortical segmentation using one of FreeSurfer’s default atlas parcellations (version 5.3.0; https://freesurfer.net), Desikan-Killiany Atlas (Desikan et al., 2006); imported the individual anatomical parcellations to Brainstorm; and averaged source signals within each of the atlas’ 68 anatomically distinct brain regions. The atlas was chosen based on the previous study (Farahibozorg, Henson, & Hauk, 2018), reporting that the optimal size of parcellation to capture independent EEG signals is around 70 regions. To mitigate source-leakage, we excluded 14 regions whose signals were collinear with other regions based on a QR decomposition (*qr* function in MATLAB) (Supporting Information Figure S1). The remaining 54 regional signals were detrended and bandpass filtered within canonical frequency bands: delta (1–3 Hz), theta (4–7 Hz), alpha (8–12 Hz), beta (13–25 Hz), and gamma (30–45 Hz). Next, we removed all shared signal at zero lag between the regions using a procedure that identifies orthogonal time courses maintaining the closest similarity to the original, unmodified time series (Colclough, Brookes, Smith, & Woolrich, 2015). Finally, amplitude envelopes were computed for each canonical frequency band and brain region using the Hilbert transform, and the envelopes were downsampled to 40 Hz (Baker et al., 2014; Hunyadi et al., 2019).

### HMM of Connectome States

HMM is detailed in our prior work (Jun et al., 2024). Briefly, we applied HMM (HMM-MAR toolbox; Vidaurre et al., 2016) to the region-wise EEG amplitude time series separately for each frequency band and obtained six discrete, recurring connectome states (*K* = 6). Generally, in choosing *K*, the objective is not to establish a “correct” number of states but to strike a balance between model complexity and fit, describing the dataset at a useful granularity (Quinn et al., 2018). In our prior study that informs the current work (Jun et al., 2024), we chose *K* = 6 in accord with several preceding HMM studies of EEG and MEG data (Coquelet et al., 2022; Quinn et al., 2018), and upon establishing heritability of HMM-derived dynamics, carried that choice forward to the current study.

To demonstrate that the connectome’s state sequencing is not occurring by chance, we employed a null model of simulated-state time courses for each frequency band, preserving the static covariance structure but intentionally disrupting the temporal ordering of states (Vidaurre et al., 2016). HMM was applied on each of these simulated time courses, and the connectome phenotypes (cf. below) were calculated. We confirmed that the original dataset’s nonrandom distribution of phenotypes over states represented veridical dynamics as it was absent in the simulated data (see Figure S2 in Jun et al., 2024).

### Multivariate Temporal Features of the Dynamic Connectome

The HMM-derived estimates provide a comprehensive set of multivariate temporal features that simultaneously characterized all states of the dynamic connectome. These estimates describe the temporal aspects of connectome dynamics by characterizing the sequence of connectome states, namely, the trajectory of the connectome through state space. For each subject, we calculated the Fractional Occupancy (the proportion of total time spent in a given state; 1 × *K*) and Transition Probability (the probability matrix of transitioning between all possible pairs of discrete states; *K* × *K*). While Transition Probability and Fractional Occupancy are not fully independent measures, they contain nonoverlapping information about connectome dynamics (cf. Jun et al., 2024). Notably, our prior work demonstrated strong genetic effects specifically in these two temporal phenotypes, both in the infraslow range using fMRI (Jun et al., 2022) and in the rapid range of dynamics using EEG (Jun et al., 2024).

### Association Between Dynamic Trajectories of States and Cognitive Performance

In our previous work (Jun et al., 2024), we employed two distinct methods (i.e., analysis of covariance and genetic variance component modeling approaches) to assess the heritability of temporal phenotypes across frequency bands. Our results from these approaches provided converging evidence for substantial heritability of Transition Probability in the theta, alpha, and gamma bands, as well as Fractional Occupancy in the beta and gamma bands. Therefore, in the present study, we investigated behavioral associations of these heritable connectome dynamics phenotypes using CCA. CCA tests for linear relationships (or “mode”) between two sets of variables (Hotelling, 1936), and as such as been previously applied to the static connectome and HMM-derived state transitions (Eichenbaum et al., 2021; Jun et al., 2022; Smith et al., 2015; Vidaurre et al., 2017). Here, we trained CCA on the dimensionality-reduced temporal phenotypes of source-space EEG connectome dynamics (U canonical variate matrix detailed below) and cognitive measures (V canonical variate matrix detailed below).

To build the U matrix, we integrated heritable phenotypes of rapid connectome dynamics from all frequency bands. Specifically, these phenotypes encompassed (a) the off-diagonals of the *K* × *K* Transition Probability matrix (30 dimension) from the theta and alpha bands, (b) 1 × *K* Fractional Occupancy (6 dimensions) from the beta band, and (c) a composite of off-diagonals of the *K* × *K* Transition Probability and 1 × *K* Fractional Occupancy (36 dimensions) from the gamma band. All variables were normalized (*z*-scored) before dimensionality reduction (Wang et al., 2020). For each band separately, principal component analysis (PCA; Jolliffe, 2014) was applied on the above-described set of multivariate variables, and only PCs with eigenvalues exceeding 1 were retained. Subsequently, the U matrix was defined as the PCs aggregated across all bands. Detailed information of PCA on connectome phenotypes for each frequency band can be found in Supporting Information Figure S2.

**Figure 2.**
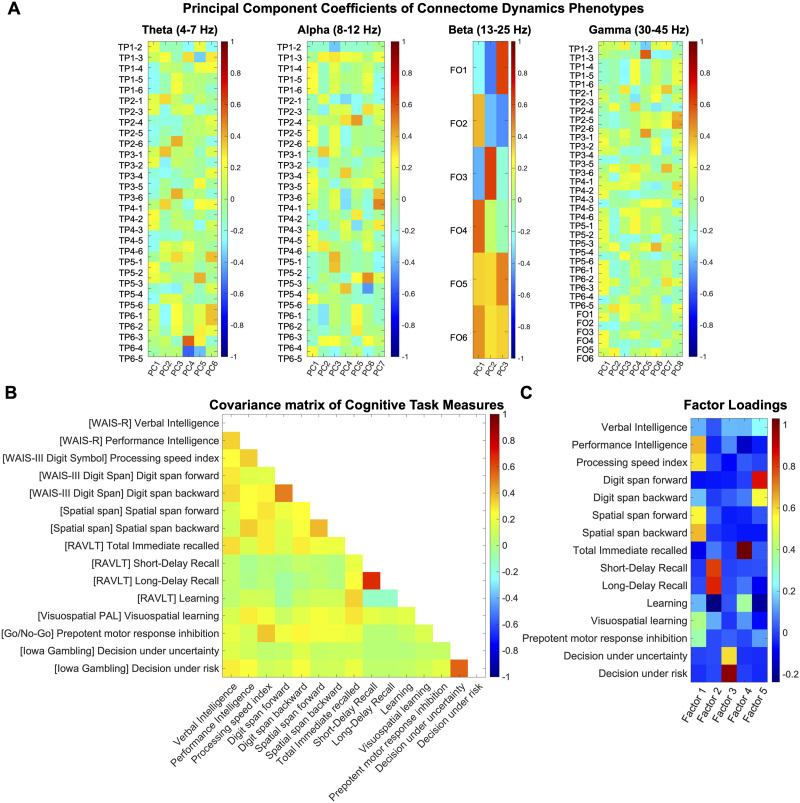
Overview of the dimensionality-reduced canonical variates. (A) The principal coefficients’ matrix displays the weight that each component of the temporal phenotypes has on each of the select number of PCs (eigenvalue > 1). (B) Empirical correlation matrix for the 15 cognitive performance measures from the Minnesota Twin Family Study (sample size *N* = 926), color-coded for Pearson’s correlation coefficient. See Supporting Information Table S1 for details of cognitive measures and tasks. (C) The factor loading matrix displays the weight that each cognitive measure has on each of the factors. FO, Fractional Occupancy; TP, Transition Probability (for example, TP1–2 denotes TP from state 1 to state 2); PC, principal component.

The V matrix encompassed 15 performance measures from eight cognitive tasks provided by the Minnesota Twin Family Study (see Supporting Information Table S1 for details of cognitive task measures). Again, measures were *z*-scored before applying dimensionality reduction. We adopted dimensionality reduction methods used for cognitive measures in prior research (Han & Adolphs, 2020; Jun et al., 2022). Specifically, we first conducted the PCA on cognitive measures to identify the number of PCs with eigenvalues surpassing 1 (Supporting Information Figure S3A). Subsequently, we applied the maximum likelihood method for factor analysis, retaining the number of factors, determined based on the preceding PCA. Notably, due to the lack of evidence for the orthogonality among the cognitive measures (as depicted in Figure 2B), we used promax oblique rotation (Hendrickson & White, 1964) to adjust them. To further enhance the reliability of our analysis, we calculated factor scores using both ridge regression (Hoerl & Kennard, 1970) and Bartlett methods (Bartlett, 1937) and found that both methods yielded comparable factor scores (Supporting Information Figure S3B). Consequently, the V canonical variate comprised the factor scores derived from the regression method and entered the subsequent CCA.

To assess the statistical significance of the identified modes of covariation, 10,000 permutations of the rows of U relative to V were performed, while maintaining the within-participant structure of the data. Then, the CCA mode was recalculated for each permutation to generate a distribution of random canonical variate pair correlation values, and each mode of covariation identified in the real data was compared with the random distribution (Smith et al., 2015; Supporting Information Figure S4). Furthermore, post hoc correlations between the modes and the cognitive factors allowed for determining the contribution of each factor to the given mode and providing further insights into the relationships between the cognitive measures and dynamic connectome features.

## RESULTS

### Connectome Phenotypes and Cognitive Measures Entering the CCA

In our prior investigation in the same cohort (Jun et al., 2024), we established heritability of *temporal* phenotypes of rapid connectome dynamics, specifically Transition Probability in theta, alpha, and gamma bands, as well as Fractional Occupancy in beta and gamma bands. Building upon these findings, we investigated the association between the heritable connectome dynamics phenotypes and cognitive measures using CCA. Prior to conducting CCA, we applied PCA to the phenotypes of each band separately. This resulted in six PCs for the theta band (accounting for 81.24% of total variance), seven for the alpha band (accounting for 86.79% of total variance), three for the beta band (accounting for 87.36% of total variance), and eight for the gamma band (accounting for 88.14% of total variance). These 24 PCs were aggregated to build a canonical variate for CCA. Figure 2A provides a comprehensive illustration of the specific contributions of connectome phenotypes to each PC. Further information of PCA on connectome phenotypes for each frequency band can be found in Supporting Information Figure S2.

As for the cognitive measures, we applied dimensionality reduction (factor analysis following PCA) and retained five cognitive factors. Figure 2C provides insights into the extent to which each cognitive measure contributed to these factors. With cautious acknowledgment that labeling reduced dimensions is suboptimal by nature, we characterize the factors as follows: Factor 1: “Visuospatial Processing” (indicated by high positive loadings across several tasks sharing visuospatial demands), Factor 2: “Verbal Memory” (indicated by high positive loadings of Short and Long Delay Recall measures from the Rey Auditory Verbal Learning Task (RAVLT)), Factor 3: “Reward-based decision-making” (indicated by high positive loadings of Iowa Gambling Task measures), Factor 4: “Verbal working memory” (indicated by the high positive loading of the Total Immediate Recall measure from RAVLT), and Factor 5: “Auditory working memory” (indicated by high positive loadings on the Wechsler Adult Intelligence Scale, third edition (WAIS-III) Digit Span task measures).

### Temporal Phenotypes of EEG Connectome Dynamics Are Associated With Cognition

**Figure 3.**
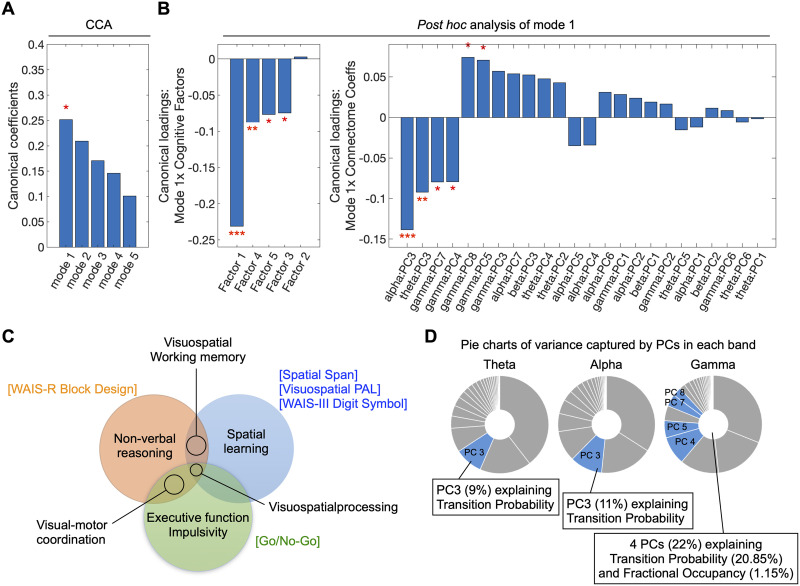
Heritable temporal dynamic connectome phenotypes are related to cognition. CCA finds the maximum linear correlation between two multidimensional canonical variates: U and V, adjusting for age and sex. Canonical variate U is defined as 24 PCs of connectome dynamics phenotypes across all frequency bands. Canonical variate V is defined as five cognitive factor loadings, each representing different cognitive domain. (A) CCA identified one significant mode of association. (B) The significant mode had contributions from four cognitive factors, namely, Factor 1: “Visuospatial Processing,” Factor 3: “Reward-based decision-making,” Factor 4: “Verbal working memory,” and Factor 5: “Auditory working memory”. On the connectome side, contributions to the significant mode came from multiple PCs spanning alpha, theta, and gamma bands. (C) The Venn diagram illustrates the relationships between different sets of cognitive task measures constructing Factor 1, which contributed significantly to the canonical mode (cf. Figure 2C). Each circle represents cognitive domains measured by the cognitive task(s) of the same color (tasks are labeled in square brackets). The overlapping areas of the circles represent the cognitive domain that is common to multiple cognitive tasks. (D) The pie charts visualize the contribution of band-specific PCs to the canonical mode. Here, each complete pie comprises the total variance in the heritable features of the given frequency band (cf. Figure 2A), and the blue areas reflect the proportion of this variance that significantly contributed to the canonical mode. *p < 0.05, **p < 0.01, ***p < 0.005.

The CCA analysis conducted on the 24 PCs of connectome phenotypes and five cognitive factors revealed a significant linear association, commonly referred to as a “mode,” after adjusting for age and sex (Figure 3A). The canonical coefficient (*r*) of the mode was found to be 0.25 with a corresponding *p* value of 0.015 (Figure 3A). The identified mode underwent nonparametric statistical significance testing (10,000 permutations), establishing significance at *p* = 0.0012 (Supporting Information Figure S4).

Subsequent post hoc correlation analyses were performed to identify differential contributions (“canonical loadings”) of cognitive factors and dynamic connectome PCs to the mode (Figure 3B). Four cognitive factors and six dynamic connectome PCs contributed to the significant mode. In terms of cognitive factors, we found that Factor 1 (“Visuospatial Processing”; canonical loadings (*r*) = −0.23, *p* = 1.11e−12) contributed the most to the significant CCA mode, followed by Factor 4 (“Verbal working memory”; *r* = −0.09, *p* = 0.007), Factor 5 (“Auditory working memory”; *r* = −0.08, *p* = 0.020), and Factor 3 (“Reward-based decision-making”; *r* = −0.07, *p* = 0.024). Given the strong loading of Factor 1 compared with the other factors, Figure 3C shows the task measures captured by this factor (cf. Supporting Information Table S1) and illustrates the involved cognitive domains along with their overlap.

Regarding connectome dynamics, we found significant contributions of PCs from theta, alpha, and gamma bands (Figure 3D): alpha PC3 (*r* = −0.14, *p* = 2.40e−05), theta PC3 (*r* = −0.09, *p* = 0.005), gamma PC7 (*r* = −0.08, *p* = 0.015), gamma PC4 (*r* = −0.08, *p* = 0.016), gamma PC8 (*r* = 0.07, *p* = 0.024), and gamma PC5 (*r* = 0.07, *p* = 0.032). Interestingly, as seen in Figure 3D, the PCs predominantly captured variance related to Transition Probabilities. Specifically, PC3 from the alpha band accounted for 11% of variance in alpha band Transition Probabilities, while PC3 from the theta band explained 9% of variance in theta band Transition Probabilities. Additionally, the four PCs from the gamma band explained 22% of variance in this band’s temporal features, all but ∼1% of which pertained to Transition Probabilities. In other words, the transitions that create the specific sequencing of connectome states, rather than the states’ total duration, are at the core of the cognitive association.

For completeness, we asked whether *spatial* features of connectome dynamics, including time-varying functional connectivity patterns and topology across states, are associated with cognition. This question is especially interesting given that these spatial features were *not* found to be heritable (Jun et al., 2024). An analogous CCA analysis performed between the five cognitive factors and the nonheritable, spatial features found no significant association (see the Supplementary Results section in the Supporting Information).

## DISCUSSION

Our current interest in the link between fast, transient connectome dynamics at subsecond timescales and cognition arises from the potential of these timescales to facilitate cognitive functioning and provide endophenotypes of translational relevance. Prior fMRI work has shown that slow time-varying connectome phenotypes may serve as promising endophenotypes for cognitive abilities (Eichenbaum et al., 2021; Jun et al., 2022; Vidaurre et al., 2017). Moving beyond infraslow timescales, our recent work has established substantial genetic influence on the phenotypes of rapid EEG connectome dynamics (Jun et al., 2024). In the present study, we investigated the relationship between these heritable phenotypes—characterizing the overall occurrence and sequencing of rapid connectome states—and a broad range of cognitive task measures. Our findings suggest that electrophysiological connectome state transitions unfolding at multiple rapid speeds collectively contribute to shape cognitive abilities.

Prior work on the functional significance of rapid whole-brain state dynamics has been largely limited to diffuse scalp topographies derived from microstate analysis in EEG or MEG (Khanna, Pascual-Leone, Michel, & Farzan, 2015; Michel & Koenig, 2018). In this approach, microstates denote recurrent, spatially diffuse sensor-level topographies that transition rapidly, typically every ∼40–200 ms (Coquelet et al., 2022; Lehmann, Pascual-Marqui, & Michel, 2009). These EEG microstates were found to be predictive of interindividual variability in cognitive abilities (Kim, Duc, Choi, & Lee, 2021; Muthukrishnan, Ahuja, Mehta, & Sharma, 2016) and linked to neurodegenerative and psychiatric disorders (da Cruz et al., 2020; Hatz et al., 2015). Yet, the diffuse topographies reflected in microstates fall short of informing about connectome states that encompass spatially localized coactivations across networks of brain regions. However, recent advances in methods have significantly improved the study of rapid connectome dynamics at cognitively highly relevant timescales (Baker et al., 2014; Brookes et al., 2014; Coquelet et al., 2022; Sitnikova et al., 2020; Wirsich et al., 2020). Despite these methodological advances, the role of rapid source-space connectome dynamics in individuals’ cognitive abilities has remained elusive.

Such role of rapid source-space connectome dynamics in individuals’ cognitive abilities is likely, given that the genetic influence on rapid dynamics is comparable in magnitude to that observed for infraslow (fMRI) connectome dynamics (and the latter is known to explain cognition). Specifically, heritability explained interindividual variance in Fractional Occupancy in beta and gamma bands at 44% and 40%, respectively. Similarly, heritability of Transition Probability in the theta, alpha, and gamma bands was estimated at 38%, 63%, and 40%, respectively (Jun et al., 2024). These magnitudes are comparable with heritability estimates for fMRI-derived Fractional Occupancy (39%) and Transition Probability (43%) (Jun et al., 2022). Confirming this predicted cognitive significance, our CCA findings unveiled that the heritable phenotypes across multiple frequency bands are significantly associated with various cognitive factors. This finding not only showcases the ability of source-space EEG to capture the functional significance of rich subsecond temporal dynamics but also demonstrates that rapid connectome transitions occurring at different speeds (i.e., theta, alpha, and gamma bands) *collectively* contribute to this relationship. Interestingly, nonheritable spatial features of rapid connectome dynamics were not associated with cognition (cf. Supporting Information), contrasting the association found for heritable temporal features. This contrast highlights the central role of genetically determined dynamic connectome phenotypes, specifically temporal ones, in explaining interindividual differences in cognition.

Importantly, our data suggest that, among the temporal features, Transition Probability holds greater functional significance than Fractional Occupancy; along with the third PC of alpha and theta bands that represent the differential contributions of Transition Probability elements, the four gamma band PCs that contributed to the significant mode of association captured largely variance in Transition Probabilities (Figure 3D). This is likely due to the fact that Transition Probability captures the temporal transition patterns that affect the specific sequencing of states, depicting the dynamic changes in interactions between brain regions. These dynamic patterns likely underlie a diverse functional repertoire in the brain (Deco, Jirsa, & McIntosh, 2013; Petersen & Sporns, 2015) relevant for a wide range of behavioral and cognitive outcomes (Cohen, 2018; Kucyi et al., 2018).

The most substantial contribution to the connectome-cognition association emerged from the “Visuospatial Processing” factor (*r* = −0.23) and PC3 of the alpha band (*r* = −0.14). This observation may be attributed to the extensive role of the alpha rhythm in shaping perception and cognition through the modulation of cortical excitability and subsequent signal processing of neural populations. In particular, alpha oscillation power is known to impact performance in tasks involving visuospatial processing, attention, working memory, and other higher order cognitive control functions (Klimesch, 2012; Mathewson et al., 2012; Palva & Palva, 2011; Sadaghiani & Kleinschmidt, 2016). Alternatively, or additionally, this observation may arise because visuospatial processing is heavily represented among the neurocognitive measures included in our study. As depicted in Figure 2C, the “Visuospatial Processing” factor contains high positive loadings from most of the measures (five out of eight tasks) and encompasses various other cognitive functions, including visuospatial working memory, visual–motor coordination, and other higher order cognitive domains (Figure 3C). Moreover, the substantial heritability effect size of the alpha band phenotype (63%) relative to other connectome dynamics phenotypes (38% ∼ 44%) (Jun et al., 2024) may further influence this finding.

Interestingly, the effect size of this behavioral association with rapid connectome dynamics (*r* = 0.25, *p* = 0.015) is similar to that with slow connectome dynamics (*r* = 0.23, *p* = 5.41e−11; Jun et al., 2022). While direct comparisons between these findings are not feasible due to different populations and distinct sets of cognitive measures included in each study, it is interesting to note that the “Language” factor contributed the most (*r* = −0.21) to the relationship with slow connectome dynamics, whereas the “Visuospatial Processing” factor contributed the most (*r* = −0.23) to the relationship with rapid connectome dynamics (Figure 3B).

Our study is subject to several limitations and methodological considerations. While we defined connectome features separately for canonical frequency bands, this approach does not assume or necessitate the bands to be discretely separable or oscillatory in nature. The approach is equally compatible with the view that the bands represent aperiodic electrophysiological processes at different speeds within a larger 1/*f* spectrum (He, 2014). Further, we defined the boundaries of the frequency bands according to common conventions in the field rather than according to the individual subjects’ power spectrum. While defining the bands individually may strengthen the observed associations to cognition, the nonindividualized approach should not invalidate the current findings. In addition, we have reduced the dimensionality of the connectome phenotypes using PCA. PCA can help extract the structure underlying high-dimensional data, in this case, by identifying the principal axes of variance from the numerous Fractional Occupancy and Transition Probability values. However, PCA may fall short in capturing the true generative structures of the data, potentially identifying illusory structures, particularly when its assumptions of orthogonality and linearity are not met (Dyer & Kording, 2023; Shinn, 2023). As a result, while the application of PCA allowed us to obtain an interpretable quantification of the variance contributing to the cognitive association (Figure 3D), this variance may not reflect the most accurate structure in the high-dimensional dynamic features. Other considerations concern the choice of connectome features and cognitive measures. While our study incorporated all cognitive task measures available from MCTFR, the cognitive factors included in our study are not diverse enough to adequately cover other cognitive domains, such as cognitive flexibility (switching) or language. We hope that the current work motivates future source-space MEG/EEG investigations of other cognitive domains. Another crucial aspect is that we investigated only a few hypothesis-driven connectome phenotypes (i.e., Transition Probability and Fractional Occupancy in specific bands) in the present study. These phenotypes were selected based on a preceding study, which stands as the sole investigation exploring the genetic impact on the time-varying characteristics of rapid connectome dynamics in source-space. However, it is likely that other dynamic features of the electrophysiological connectome may be individually specific and affect cognitive abilities. Future studies with sample sizes and analyses appropriate for more exploratory approaches may address a more extensive list of dynamic connectome features and behavioral measures.

Together, our findings establish that rapid, subsecond transitions between whole-brain connectome states—most notably, the states’ sequencing in alpha, theta, and gamma bands—are associated with cognitive performance including visuospatial processing abilities. This evidence substantially extends previous associations of infraslow dynamics and cognition (Eichenbaum et al., 2021; Jun et al., 2022; Vidaurre et al., 2017) to the rapid timescales at which most cognitive processes unfold. In light of the heritability of Transition Probabilities in resting-state electrophysiology (Jun et al., 2024), our findings position these connectome features as potential endophenotypes for cognitive abilities. Such endophenotypes can delineate the neurobiological (specifically connectome-based) mechanisms via which genetics can shape cognitive abilities. Further, the identified connectome phenotypes may be of clinical relevance. Infraslow (fMRI-derived) connectome dynamics are implicated in numerous psychiatric and neurological conditions (Preti, Bolton, & Van De Ville, 2017) and explain cognitive abilities across different diagnostic categories (Zhu et al., 2021). However, the role of rapid electrophysiological connectome dynamics in cognitive and mental health is largely unexplored. Addressing their untapped potential, the identified phenotypes may be explored as connectome-based biomarkers of cognitive functioning and dysfunction with cost-efficient EEG.

## ACKNOWLEDGMENTS

We thank Dr. Andre Altmann for his extensive guidance in analytic approaches and Drs. Jonathan Wirsich and Thomas Alderson for their guidance in data preprocessing. Computational resources for this work were provided by the Minnesota Supercomputing Institute at the University of Minnesota Informatics Institute. The Center for Magnetic Resonance Research (supported by Grant Nos. NIBIB P41 EB027061 and 1S10OD017974-01) at the University of Minnesota provided resources that contributed to the MRI-related results reported within this article. The original data collection of the data analyzed in this paper was funded by NIH grants R37 DA05147 and R01 DA036216. This work was partly supported by the National Institute for Mental Health (1R01MH116226 to Sepideh Sadaghiani).

## SUPPORTING INFORMATION

Supporting information for this article is available at https://doi.org/10.1162/netn_a_00390.

## AUTHOR CONTRIBUTIONS

Suhnyoung Jun: Conceptualization; Formal analysis; Investigation; Methodology; Writing – original draft. Stephen Malone: Data curation. Thomas H. Alderson: Methodology. Jeremy Harper: Methodology. Ruskin H. Hunt: Methodology. Kathleen M. Thomas: Methodology. Sylia Wilson: Data curation. William Iacono: Funding acquisition; Project administration. Sepideh Sadaghiani: Conceptualization; Funding acquisition; Methodology; Project administration; Supervision; Writing – original draft.

## DATA AVAILABILITY STATEMENT

The derivative data used in the study are available upon reasonable request.

## FUNDING INFORMATION

The University of Minnesota Center for Magnetic Resonance Research, National Institute of Biomedical Imaging and Bioengineering (https://dx.doi.org/10.13039/100000070), Award ID: NIBIB P41 EB027061. The University of Minnesota Center for Magnetic Resonance Research, National Institute of Biomedical Imaging and Bioengineering (https://dx.doi.org/10.13039/100000070), Award ID: NIBIB P41 1S10OD017974-01. The University of Minnesota Center for Magnetic Resonance Research, National Institutes of Health (https://dx.doi.org/10.13039/100000002), Award ID: R37 DA05147. The University of Minnesota Center for Magnetic Resonance Research, National Institutes of Health (https://dx.doi.org/10.13039/100000002), Award ID: R01 DA036216. Sepideh Sadaghiani, National Institute of Mental Health and Neurosciences (https://dx.doi.org/10.13039/100019274), Award ID: 1R01MH116226.

## Supplementary Material



## TECHNICAL TERMS

Connectome states: Discrete functional brain connectivity patterns recurring over time.
Hidden Markov model (HMM): Statistical model for sequences with unobserved states.
Endophenotypes: Intermediate heritable traits that can help in understanding the genetic basis of complex disorders.
Source-leakage: This phenomenon where the estimated sources of neural activity are affected by artifacts or signals that are not of interest.
Phenotypes: Observable traits influenced by genetics and environment.
Canonical correlation analysis (CCA): Technique to explore linear association between two sets of variables.
Multivariate dynamic connectome phenotypes: Phenotypes that comprehensively represent the time-varying changes of all states.
Orthogonalization: Process used to isolate specific brain activity from noise or other interfering signals.
Factor analysis: Method to extract latent cognitive factors across numerous task measures, overcoming the influence of tasks’ unreliability and measurement impurity.
