# Supplementary material for: Cognitive abilities are associated with rapid dynamics of electrophysiological connectome states
Source: Netw Neurosci. 2024 Dec 10;8(4):1089–104. doi: 10.1162/netn_a_00390 (PMC11674572; doi:10.1162/netn_a_00390)
Supplement: Supplementary file 1 [file netn-8-4-1089-s001.pdf]

Supplementary Information

Supplementary Results

The core observation of the main manuscript is that temporal phenotypes of rapid, EEG-derived connectome dynamics are associated with cognitive performance. Here, we add a supplementary analysis to investigate whether the observed association is specific to the investigated temporal features, or whether it extends to a set of spatial features. While the temporal features describe states' occupancy and sequencing, the spatial features characterize the states' connectivity pattern.

Regarding the spatial features, our prior investigation in the same cohort (Jun et al., 2024) found no evidence for heritability in any frequency band. Specifically, the spatial features comprised two time-varying aspects, each defined in a multivariate manner (6-dimensional across six states) paralleling the approach for the temporal features. Firstly, time-varying functional connectivity (FC) was assessed as state-by-state FC averaged within a data-driven cluster that showed significant FC difference across the connectome states. Secondly, time-varying Modularity was used to quantify the degree of topological segregation-integration of all individual states. The heritability investigation for the multivariate, time-varying spatial features are detailed in (Jun et al., 2024). None of these spatial features was found to be heritable. While we acknowledge that other time-varying spatial features beyond those investigated in our work may be heritable, the lack of evidence for heritability in a broad set of spatial features (see supplementary materials of (Jun et al., 2024) for heritability investigations of additional exploratory spatial features) strongly contrasts the impact of genetics found for time-varying temporal features.

In order to extend our CCA analysis to the above-characterized recurrent spatial patterns, we performed canonical correlation analysis (CCA) to investigate the association between the (non-heritable) dynamic spatial features and cognitive measures. This analysis was analogous to the approach applied to the temporal features in the main manuscript. Specifically, prior to conducting CCA, we applied PCA to the above-described multivariate spatial features (i.e., $1 \times K$ time-varying FC and $1 \times K$ time-varying Modularity; where K stands for 6 states) separately for each frequency band. This resulted in 5 principal components (PCs) with an eigenvalue >1 for the delta band (together accounting for 61.31% of total variance), 5 PCs for the theta band (accounting for 55.93% of total variance), 5 for the alpha band (accounting for 55.61% of total variance), 5 for the beta band (accounting for 55.75% of total variance), and 5 for the gamma band (accounting for 51.28% of total variance). These 25 PCs were aggregated to build a canonical variate for CCA. The CCA analysis conducted on the 25 PCs of connectome spatial features and 5 cognitive factors (see main text, **2.7. Multivariate temporal features of the dynamic connectome**) revealed no significant mode, i.e., no linear association. Thus, the non-heritable spatial features of rapid connectome dynamics investigated in our study do not explain inter-individual differences in cognition, contrasting the cognitive association found for heritable temporal features.

Supplementary Tables

Table S1. Cognitive Task Measures from Minnesota Twin Family Study

Cognitive Assessments	Task Description	
	**Variable Names**	**Measured Performance**
WAIS-R Vocabulary and Block Design (Wechsler & De Lemos, 1981)	Vocabulary Subtest: The examiner asks the test taker to define each word in a series of words presented orally. Block Design Subtest: The test taker is presented with a set of blocks with surfaces that are either red, white or half red and half white. The examiner presents a series of abstract patterns, which the test taker is required to replicate using the blocks. Subsequent trials involve an increasing number of blocks and increasingly difficult patterns.	
	Verbal intelligence	The accuracy and depth of the word definitions. Verbal reasoning, verbal concept formation and expressive language.
	Nonverbal intelligence	Number of designs correctly replicated within the time limit. Non-verbal reasoning and visual-spatial abilities.
WAIS-III Digit Symbol (Wechsler, 1997)	Digit Symbol Coding: On the top of the page, a key with two rows (top: one-to-nine digits, bottom: a symbol for each digit) is presented. Test takers copy the symbols into spaces below a row of numbers according to the key. Symbol Search: Two target symbols appearing on the left of a row are sought among an array of five symbols on the right. Test takers respond by either marking the identical symbol, or a “no” box (if the matching symbol is not present in the array).	
	Processing Speed Index	Sum of subtest scores (Digit Symbol Coding and Symbol Search Tasks). Ability to scan and process visuospatial stimuli; processing speed; attention.
Rey Auditory Verbal Learning Test (Rey, 1958)	Trials 1 to 5: A sequence of 15 words (List A) is read aloud by an examiner, and the test taker is immediately asked to recall as many words on the list as possible.<br>List B: After that, a different set of 15 words (List B) is read to the test taker, who then is immediately asked to recall the words.<br>Trial 6: Immediately after List B, the test taker is asked to recall the words from List A.	

	Trial 7: Recall, unwarned, the words from List A after a delay of 30 minutes after Trial 6, during which time the test taker performs a series of nonverbal tasks.	
	Total immediate recall	Sum of words immediately recalled after the List A is read to the test taker across Trial1 ~ Trial 5
	Short-delay recall	Trial 6 recall performance – Trial 5 recall performance
	Long-delay recall	Trial 7 recall performance – Trial 5 recall performance
	Learning over trial	Trial 5 – Trial 1
Visuospatial Paired Associates Learning (Sahakian et al., 1988)	Boxes are displayed on the periphery of the computer screen and a subset of them are "opened" in a randomized order, revealing an abstract pattern inside. Each pattern is then displayed in the middle of the screen, one at a time, and the test taker must select the box in which the pattern was originally located. If the test taker selects the wrong box, the trial is repeated: the boxes are opened in sequence again and then each pattern is displayed in the center of the screen in turn and the test taker must indicate the box in which the pattern was originally located. This repeats until the test taker successfully indicates the original location of all patterns in the sequence. The number of boxes (the length of the sequence of boxes) varies from two to eight.	
	Visuospatial Learning	Inverse value of total error numbers. Visuospatial learning and memory.
WAIS-III Digit Span (Wechsler, 1997)	The examiner presents a sequence of numbers orally, starting with two digits and progressively increasing in length to a maximum of nine. Two trials are given for each sequence length, and the test continues until the test taker fails to recall the sequence correctly on both trials.<br>Forward Digit Span: Test-takers repeat the read-out list of digits in the order presented.<br>Backward Digit Span: Test-takers repeat the read-out list of digits in reverse order.	
	Forward Digit Span	Number of correct trials. Attention and Working memory.
	Backward Digit Span	Number of correct trials. Working memory capacity and Executive function.
Spatial Span Task (Sahakian & Owen, 1992)	Analogous to Digit Span but in the visual-spatial domain to assess memory for figural patterns. An array of 10 white boxes is displayed in arbitrary locations on a computer screen. The stimulus sequences start with a span of two: two boxes change color, one at a time, after which the subject is required to reproduce the sequence by pointing at each box in sequence with a computer mouse. Sequences become progressively longer, with a maximum of nine.	

	Forward Spatial Span: Test-takers recall the sequence of box locations in the order in which they occurred. Backward Spatial Span: Test-takers recall the sequence of box locations in reverse order.	
	Forward Spatial Span	Number of correct trials. Attention and Working memory.
	Backward Spatial Span	Number of correct trials. Working memory capacity and Executive function.
Go/No-Go task (Harper et al., 2018)	Two white letters were alternately centrally presented on a black background, and participants were instructed to make a button press whenever a letter followed a different letter (go; e.g., second letter in the sequence X-Y), but withhold their response when the letter was identical to the preceding letter (no-go; e.g., third letter in the sequence X-Y-Y).	
	Prepotent motor inhibition	Inverse value of false alarm rate (%). Ability to inhibit prepotent motor response.
Iowa Gambling Task (Brand et al., 2007)	Test takers are presented with an image of four decks of cards on a computer screen. Each deck contains a mixture of gains and losses. Test takers are instructed to choose cards from these decks without knowing their characteristics to accumulate as much money as possible. A selection from each deck earns money, but some selections lost money. The amount of money earned and lost, and the frequency of losses varies across the decks. The test taker receives feedback after each selection in the form of the amount of money gained and lost, as well as a running total of their "earnings."	
	Decision under Uncertainty	Decision-making under uncertainty. Ability to learn from feedback and adjust their choices based on learning the risk and reward outcomes of the different decks This is defined as the number of selections from net-advantageous decks relative to net-disadvantageous decks during the first 60 trials (out of 100).
	Decision under Risk	Decision-making under risk. Ability to maximize "earnings" after experience with the relative amount of gains (reward) and frequency and magnitude of risk (losses). This is defined as the number of selections from net-advantageous decks relative to net-disadvantageous decks during the last 40 trials.

Note: The PAL, spatial span, go/no-go and IGT tasks were programmed using E-Prime software, version 2.0 (Psychology Software Tools, Pittsburgh, PA). WAIS-R; Wechsler Adult Intelligence Scale-Revised (WAIS-R).

Supplementary Figures

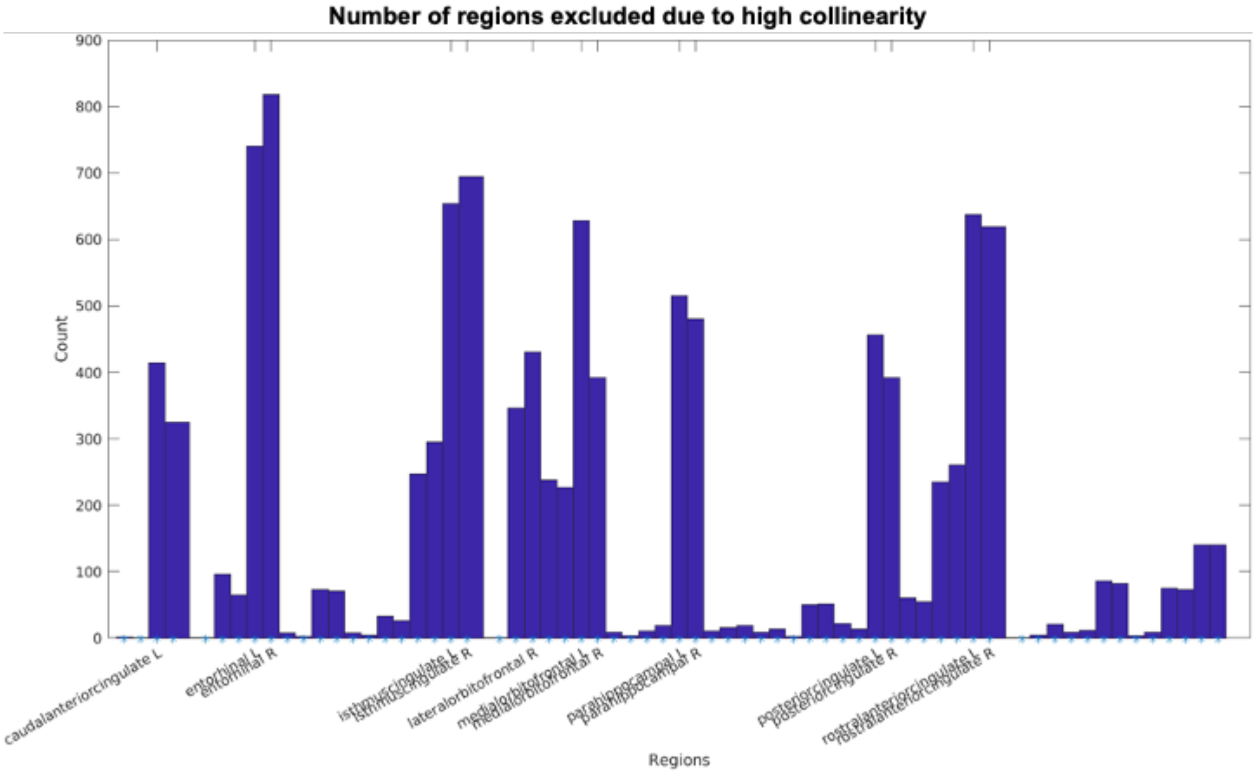

Figure S1. Top 14 regions with high collinearity. To mitigate the source-leakage confound, caused by the blurring of point dipole sources and the spreading of signals across neighboring regions, we excluded regions, whose extracted signals were found to be highly collinear with others based on *qr* function in Matlab. Subsequently, 14 regions were excluded from the investigation: bilateral ‘*rostralanteriorcingulate’*, bilateral '*posteriorcingulate*', bilateral '*parahippocampal*', bilateral '*medialorbitofrontal'*, bilateral '*isthmuscingulate*', bilateral '*entorhinal*', '*lateralorbitofrontal R*', and '*caudalanteriorcingulate L'*.

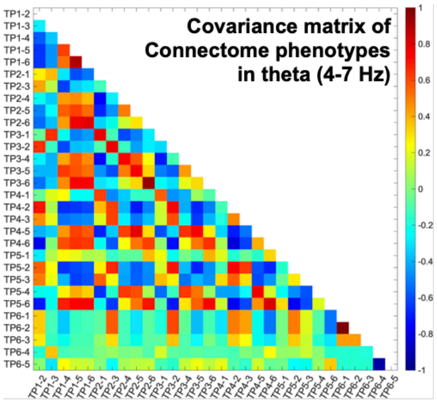
Covariance matrix of
Connectome phenotypes
in theta (4-7 Hz)

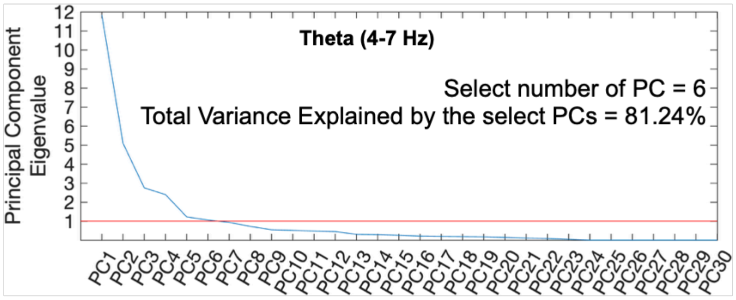
Theta (4-7 Hz)
Select number of PC = 6
Total Variance Explained by the select PCs = 81.24%
Principal Component
Eigenvalue

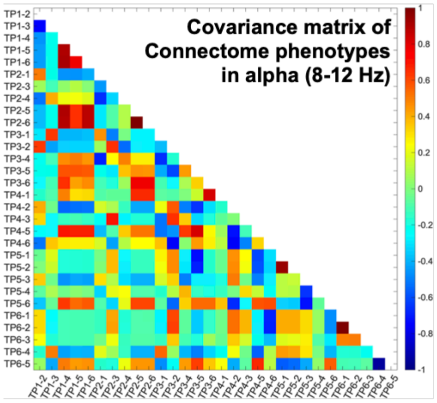
Covariance matrix of
Connectome phenotypes
in alpha (8-12 Hz)

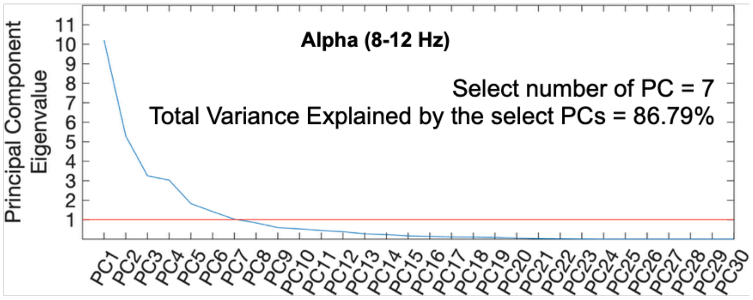
Alpha (8-12 Hz)
Select number of PC = 7
Total Variance Explained by the select PCs = 86.79%
Principal Component
Eigenvalue

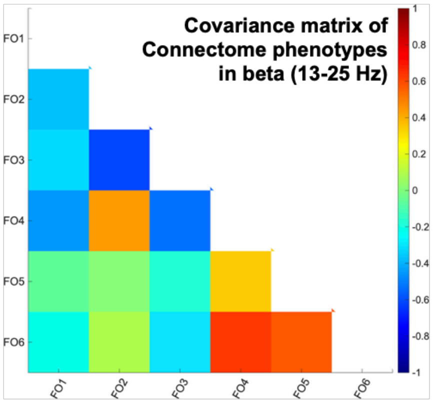
Covariance matrix of
Connectome phenotypes
in beta (13-25 Hz)
FO1
FO2
FO3
FO4
FO5
FO6

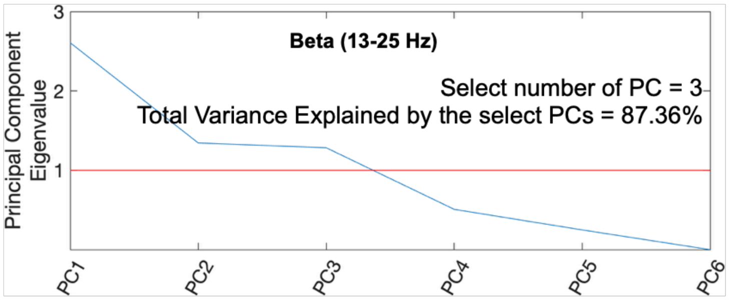
Beta (13-25 Hz)
Select number of PC = 3
Total Variance Explained by the select PCs = 87.36%
Principal Component
Eigenvalue
PC1
PC2
PC3
PC4
PC5
PC6

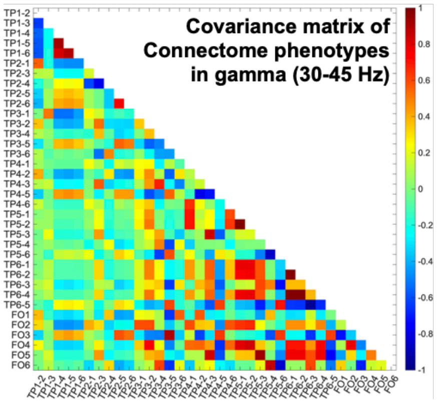
Covariance matrix of
Connectome phenotypes
in gamma (30-45 Hz)

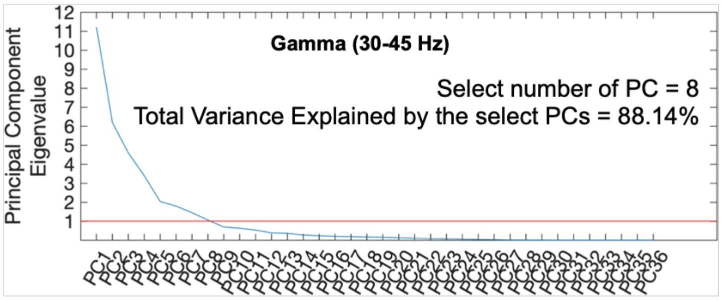
Gamma (30-45 Hz)
Select number of PC = 8
Total Variance Explained by the select PCs = 88.14%
Principal Component
Eigenvalue

Figure S2. Overview of the principal component analysis on temporal features of the dynamic connectome. (Left) Pearson's correlation matrix for the temporal features of the dynamic connectome obtained from the six-state model, color-coded for Pearson's correlation coefficient. (Right) Scree plot of the eigenvalues of principal components of the temporal connectome dynamics features for each frequency band. Red colored reference line (y = 1) indicates the cut-off value used for the present study.

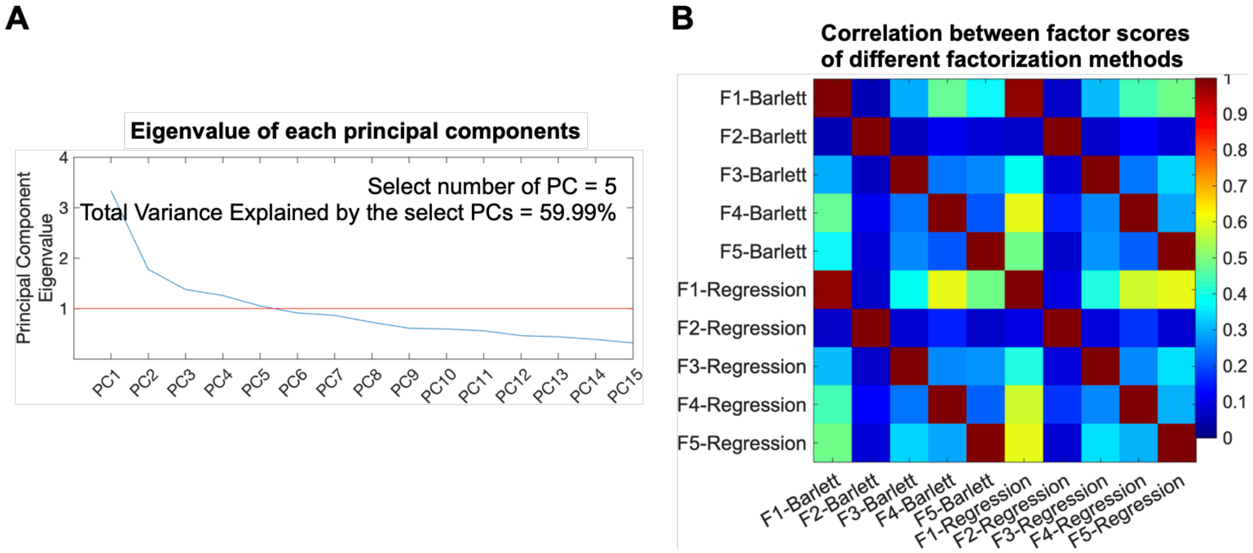

Figure S3. Overview of the cognitive dataset. (A) Scree plot of the eigenvalues of principal components obtained from the covariance matrix of the cognitive measures, where red colored reference line (y = 1) indicates the cut-off value used for the present study. **(B)** Pearson's correlation matrix for two sets of factor scores derived using Bartlett method (F1-Barlett to F5-Barlett) and ridge regression method (F1-Regression to F5-Regression). PC: principal component, F: Factor.

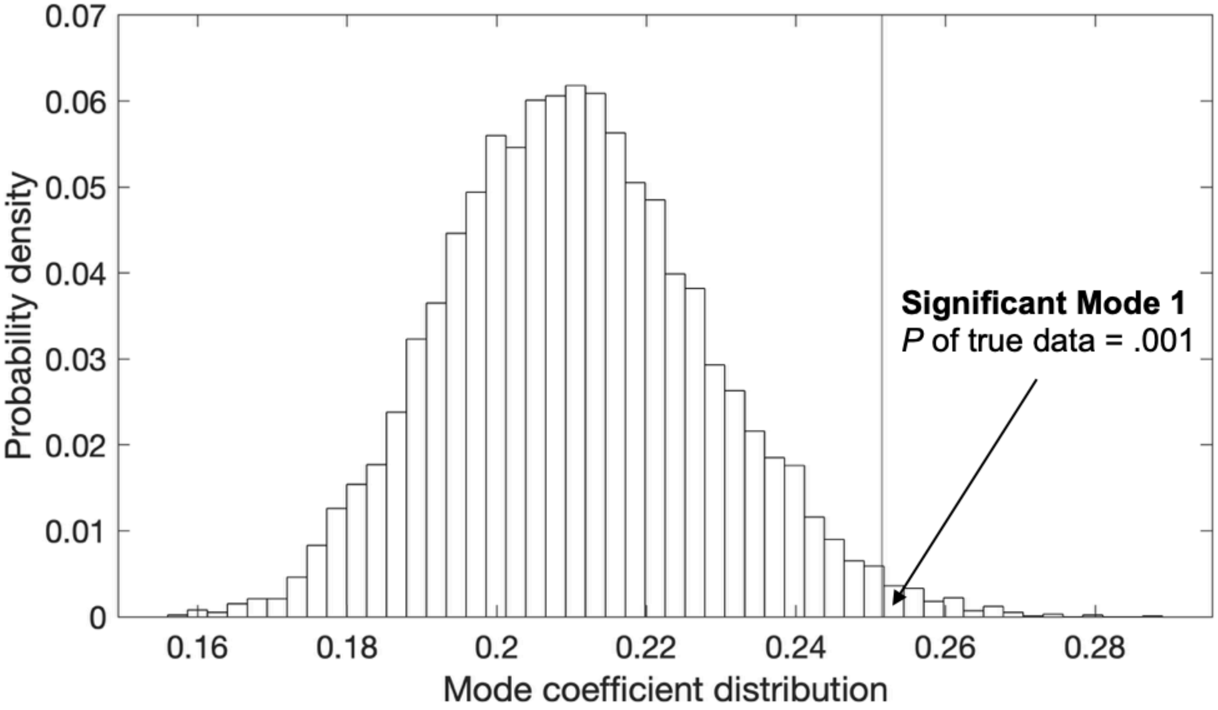

Figure S4. The distribution of random canonical variate pair correlation values. The histogram shows the distribution of random canonical variate pair correlation values from 10,000 permutations of the rows of U relative to V were performed, while maintaining the within-participant structure of the data. The mode of covariation identified in the real data, compared against the permutations, showed significance at $p = .0012$.

Supplementary References

Brand, M., Recknor, E. C., Grabenhorst, F., & Bechara, A. (2007). Decisions under ambiguity and decisions under risk: Correlations with executive functions and comparisons of two different gambling tasks with implicit and explicit rules. *Journal of Clinical and Experimental Neuropsychology*, *29*(1), 86–99. https://doi.org/10.1080/13803390500507196

Harper, J., Malone, S. M., & Iacono, W. G. (2018). Impact of alcohol use on EEG dynamics of response inhibition: A cotwin control analysis. *Addiction Biology*, *23*(1), 256–267. https://doi.org/10.1111/adb.12481

Jun, S., Malone, S., Iacono, W. G., Wilson, S., Sadaghiani, S., & Harper, J. (2024). *Rapid dynamics of electrophysiological connectome states are heritable* (p. 2024.01.15.575731). bioRxiv. https://doi.org/10.1101/2024.01.15.575731

Rey, A. (1958). *L'examen clinique en psychologie. [The clinical examination in psychology.]* (p. 222). Presses Universitaries De France.

Sahakian, B. J., MORRIS, R. G., EVENDEN, J. L., HEALD, A., LEVY, R., PHILPOT, M., & ROBBINS, T. W. (1988). A COMPARATIVE STUDY OF VISUOSPATIAL MEMORY AND LEARNING IN ALZHEIMER-TYPE DEMENTIA AND PARKINSON'S DISEASE. *Brain*, *111*(3), 695–718. https://doi.org/10.1093/brain/111.3.695

Sahakian, B. J., & Owen, A. M. (1992). Computerized assessment in neuropsychiatry using CANTAB: Discussion paper. *Journal of the Royal Society of Medicine*, *85*(7), 399–402.

Wechsler, D. (1997). *Wechsler Adult Intelligence Scale—Third Edition* [dataset]. https://doi.org/10.1037/t49755-000

Wechsler, D., & De Lemos, M. M. (1981). *Wechsler adult intelligence scale-revised (Vol. 1)*. New York: Psychological Corporation.
